# H5N1 Influenza a Virus Replicates Productively in Pancreatic Cells and Induces Apoptosis and Pro-Inflammatory Cytokine Response

**DOI:** 10.3389/fcimb.2018.00386

**Published:** 2018-11-06

**Authors:** Caiyun Huo, Kai Xiao, Shouping Zhang, Yuling Tang, Ming Wang, Peng Qi, Jin Xiao, Haiyan Tian, Yanxin Hu

**Affiliations:** ^1^Key Laboratory of Animal Epidemiology of Ministry of Agriculture, College of Veterinary Medicine, China Agricultural University, Beijing, China; ^2^College of Animal Science and Veterinary Medicine, Henan Institute of Science and Technology, Xinxiang, China; ^3^Key Laboratory of Veterinary Bioproduction and Chemical Medicine of the Ministry of Agriculture, Zhongmu Institutes of China Animal Husbandry Industry Co., Ltd., Beijing, China

**Keywords:** H5N1 influenza A virus, pancreatic cells, pathogenesis, apoptosis, inflammatory response

## Abstract

The inflammatory response and apoptosis have been proved to have a crucial role in the pathogenesis of the influenza A virus (IAV). Previous studies indicated that while IAV commonly causes pancreatitis and pancreatic damage in naturally and experimentally infected animals, the molecular mechanisms of the pathogenesis of IAV infection are less reported. In the present study, we showed for the first time that both avian-like (α-2,3-linked) and human-like (α-2,6-linked) sialic acid (SA) receptors were expressed by the mouse pancreatic cancer cell line PAN02 and the human pancreatic cancer cell line PANC-1. Using growth kinetics experiments, we also showed that PAN02 and PANC-1 cells supported the productive replication of the H5N1 highly pathogenic avian influenza while exhibited the limited replication of IAV subtypes H1N1 and H7N2 *in vitro*. The *in vivo* infection of H5N1 in pancreatic cells was confirmed by the histopathological and immunohistochemical staining of pancreas tissue from mice. Other than H1N1 and H7N2, severe damage and extensive positive signals were observed in pancreas of H5N1 infected mice. All three virus subtypes induced apoptosis but also triggered the infected PAN02 and PANC-1 cells to release pro-inflammatory cytokines and chemokines including interferon (IFN)-α, IFN-β, IFN-γ, chemokine (C-C motif) ligand 2 (CCL2), tumor necrosis factor (TNF)-α, and interleukin (IL)-6. Notably, the subtypes of H5N1 could significantly upregulate these cytokines and chemokines in both two cells when compared with H1N1 and H7N2. The present data provide further understanding of the pathogenesis of H5N1 IAV in pancreatic cells derived from humans and mammals and may also benefit the development of new treatment against H5N1 influenza virus infection.

## Introduction

H5N1 avian influenza A virus (IAV), one serious infectious etiology in respiratory disease, brings great panic and threat to the breeding industry and humans due to its severe morbidity and high fatality rates. Some reports have suggested that severe lung injury is triggered by cytokine dysregulation, which is referred to as the “cytokine storm” and is a result of an excessive inflammatory response (López Cde et al., 2009; Cheng et al., 2011). This dysregulation makes a significant contribution to the high mortality of H5N1 influenza(Cheung et al., 2002; de Jong et al., 2006; Us, 2008; Cillóniz et al., 2009; Tisoncik et al., 2012). In addition to the respiratory systems, IAV infection can also affect other organs of the digestive and nervous systems including the spleen, liver, small intestine and brain(Leon'teva et al., 1989; Sharkova et al., 2008; Chaves et al., 2014; Zhang et al., 2015).

The pancreas is an important digestive gland, which consists of an exocrine and an endocrine gland that produces digestive enzymes and hormones. The exocrine cells of the pancreas contain many rounded acini and ducts, while the endocrine cells form the pancreatic islets. Several studies have shown that the IAV has a tropism for the pancreas, which can cause mammals and humans to suffer from acute pancreatitis and pancreatic damage following IAV infection (Calore et al., 2011; Kasloff et al., 2014). Microscopic lesions and viral antigens have been observed most frequently in the pancreas following IAV infection, where necrosis of pancreatic cells and infiltration of inflammatory cells are apparent(Yingst et al., 2006; Pantin-Jackwood and Swayne, 2007; Reperant et al., 2012; Zhang et al., 2015; Haider et al., 2017). However, the pathogenic mechanism remains to be determined.

Influenza viruses can initiate infection and viral replication by binding to neuraminic acids (sialic acids, SA) that is located on the surface of cells. One study showed that α-2,3-SA and α-2,6-SA receptors usually located on ciliated and non-ciliated cells respectively(Matrosovich et al., 2004). In general, α-2,3-linked SA receptors are preferentially recognized by avian influenza viruses, while α-2,6-linked SA receptors are recognized by human influenza viruses (Van Poucke et al., 2010; Raman et al., 2014). Therefore, the type of SA receptor on cellular surface maybe largely determine the influenza virus tropism (Suzuki et al., 2000). Our previous studies demonstrated that both linkages are expressed in certain cells of the respiratory and gastrointestinal tracts of humans and mammals (Zhang et al., 2015; Meng et al., 2016). Nevertheless, little information was available about SA receptor location on pancreatic cells derived from different origins.

Differ from necrosis, apoptosis is a genetically controlled process, which occurs in lots of pathogenic processes such as bacterial infection, viruse infection and cancer (Elmore, 2007; Ouyang et al., 2012). Chromatin condensation and eventually the apoptotic body formation are taken place during the apoptosis. It is demonstrated that the influenza virus could promote different cells apoptosis (Ito et al., 2002; Liu et al., 2014). The role of apoptosis is considered to be complex during IAV infection. On one hand, apoptosis induced by influenza virus infection is crucial for hosts to defense against invading viruses. On the other hand, it can facilitate the replication and spread of virus during the IAV infection (Herold et al., 2012). Apoptosis has also been involved in the symptoms of influenza virus infection that includes tissue damage (Brydon et al., 2005). In this way, apoptosis and the virus-induced hyperproduction of cytokines that occurs during the inflammatory response play a definitive role in the pathogenesis of influenza. Understanding the mechanisms of apoptosis and the pro-inflammatory response that are initiated following IAV infection would provide valuable information for development of novel therapeutic aids.

During IAV infection, viral double-stranded RNA (dsRNA) could be recognized through several pattern-recognition receptors (PRRs), such as Toll-like receptor 3 (TLR3), cytolytic RNA helicases retinoic acid-inducible gene I (RIG-I), and melanoma differentiation-associated gene 5 (MDA-5) (Yu and Levine, 2011; Tang et al., 2014). After TLR3, RIG-I and/or MDA-5 are stimulated with their respective agonist, these pathways will activate the transcription factor nuclear factor (NF)-κB. Then the production of inflammatory cytokines and chemokines can be induced, while interferon regulatory factor (IRF) 3 and/or 7 will be activated and induce the type I interferons (IFNs) secretion (Takeuchi and Akira, 2009). To date, however, little reports have evaluated the role of these signaling pathways in the process of IAV infection in pancreatic cells.

Here we demonstrated that both pancreatic cell lines express α-2,3- and α-2,6-linked SA receptors that initiate H5N1 infection. Furthermore, PAN02 and PANC-1 cells supported the productive replication of the H5N1 highly pathogenic avian influenza while exhibited the limited replication of IAV subtypes H1N1 and H7N2 *in vitro*. The infection of H5N1 in pancreatic cells was confirmed by the histopathological and immunohistochemical staining of pancreas tissue from mice. We also examined the expression of some molecules associated with apoptosis and the inflammatory response in IAV infected pancreatic cells. Our results are the first to demonstrate that the H5N1 IAV can induce apoptosis in pancreatic cells. Besides, TLR3, RIG-I and MDA-5 signaling pathways are likely involved in the process of inflammatory response of PAN02 and PANC-1 cells. Significantly, PAN02 and PANC-1 cells mediated substantial hyperinduction of pro-inflammatory cytokines and chemokines following H5N1 IAV infection. Collectively, our findings are the first to address the molecular mechanism of H5N1 IAV invasion and activation in pancreatic cells, which will contribute to a greater understanding of H5N1 IAV pathogenesis.

## Materials and methods

### Viruses and cell culture

The avian influenza viruses H5N1 (A/Chicken/Henan/1/04) and H7N2 (A/Chicken/Hebei/2/02) were isolated from infected chicken flocks and propagated in the allantoic cavities of 10-day-old embryonated chicken eggs for 24–48 h at 37°C. The working stocks of human influenza virus H1N1 (A/WSN/33) were generated in Madin-Darby canine kidney (MDCK) epithelial cells and virus titers were determined by standard plaque assays. All experiments with H5N1 viruses were conducted in a biosafety level 3 containment laboratory approved by the Ministry of Agriculture of China.

The mouse pancreatic cell line PAN02, human pancreatic cell line PANC-1, and MDCK cells were provided and cultured as previously described (Liu et al., 2014)

### *In vitro* viral infection

Cells were seeded and viral infection was taken as previously described (Liu et al., 2014). Here, TPCK trypsin was not included in media for H1N1 culture but was added to media for plaque assays.

### *In vivo* viral infection

The procedures of viral infection *in vivo* and histopathological and immunohistochemical staining were the same as previous reference published by our team (Huo et al., 2017). Animal experiments were approved by the Animal Ethics Committee of China Agricultural University (approval number 201206078) and were performed in accordance with Regulations of Experimental Animals of Beijing Authority.

All mouse experimental protocols complied with the guidelines of the Beijing Laboratory Animal Welfare and Ethics Committee (BLAWEC), and were approved by the Beijing Association for Science and Technology (the approve ID is SYXK-2009-0423).

### *In vitro* and *in vivo* detection of the expression pattern of sialic acid receptors

The expression pattern of sialic acid receptors of cells was detected as previously described (Meng et al., 2016; Tang et al., 2018).

Representative pancreas sections from mock-treated mice were collected and were fixated in 70% ethanol and the expression pattern of SNA and MAA-I were analyzed by immunohistochemical staining (Huo et al., 2017).

### Flow cytometry

The procedures of flow cytometry were performed as previously described (Meng et al., 2016).

### Transmission electron microscopy (TEM)

The methods of TEM were performed as previously described (Meng et al., 2016).

### Real-time quantitative PCR (RT-qPCR)

Expression of the viral NS1 gene, TLR3, RIG-I, MDA5, IFN-α, IFN-β, IFN-γ, chemokine (C-C motif) ligand 2 (CCL2), tumor necrosis factor (TNF)-α, and interleukin (IL)-6 was determined as previously described (Liu et al., 2014; Huo et al., 2017). Primer sequences were listed in Supplementary Material.

### Plaque assay

Plaque assays were performed as previously described (Liu et al., 2014).

### Assessment of cytopathic effects

Assessment of cytopathic effects was performed as previously described (Liu et al., 2014; Song et al., 2018).

### Terminal deoxynucleotidyl transferase-mediated dUTP-Biotin nick end labeling (TUNEL) assay

Apoptotic cells were examined with the TUNEL assay, which was performed as previously described (Liu et al., 2014).

### Flow cytometric analysis of apoptosis

The apoptotic responses of pancreatic cells were examined as previously described (Liu et al., 2014).

### Statistical analysis

Statistical analyses were taken by two-way analysis of variance (ANOVA) with the GraphPad Prism (version 5.0; GraphPad Software, San Diego, CA, USA). A *P*-value of < 0.05 represents statistically significant. Results were showed as the mean ± standard deviation of three independent experiments.

## Results

### Pancreatic cells express both α-2,3- and α-2,6-linked SA receptors

**Figure 1:**
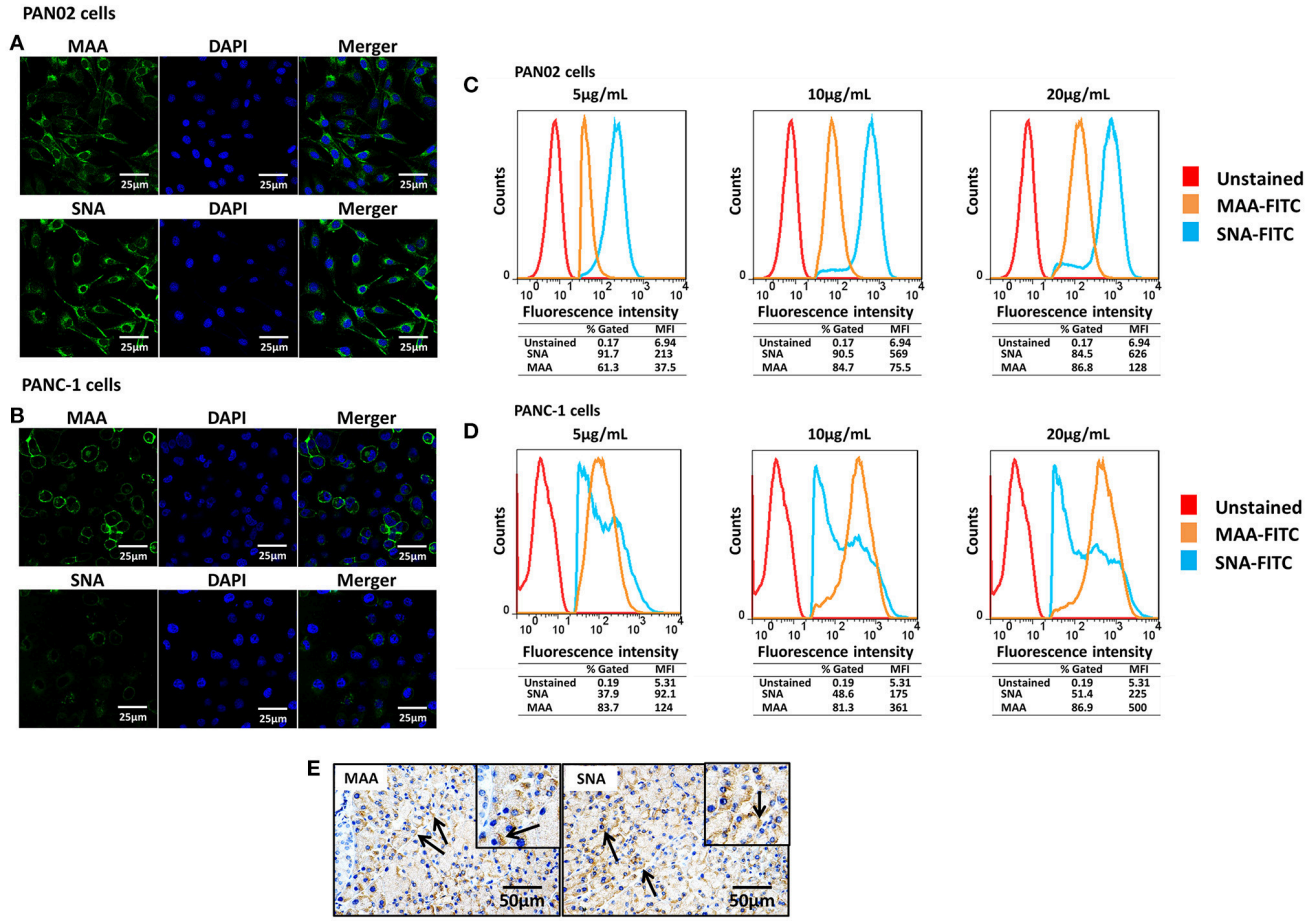
Pancreatic cells express α-2,3- and α-2,6-linked sialic acid (SA) receptors. **(A,B)** The pancreatic cell lines PAN02 and PANC-1 were placed on polylysine-coated slides and stained with fluorescein isothiocyanate (FITC)-conjugated *Sambucus nigra* bark lectin (SNA) or *Maackia amurensis* lectin I (MAA-I) (green), and 4′,6′-diamidine-2-phenylindole (DAPI; blue) for nuclei. **(C,D)** Trypsinized PAN02 and PANC-1 cells were incubated with FITC-conjugated SNA or MAA-I (concentrations from left to right are 5, 10, and 20 μg/mL) and analyzed using flow cytometry to determine the relative percentages of cells expressing α-2,3-SA (MAA, yellow) or α-2,6-SA (SNA, blue) compared to unstained cells (red). **(E)** Representative pancreas sections from mock-treated mice were analyzed by immunohistochemical staining using SNA and MAA-I antibody, respectively. Black arrows indicate positive signals.

SA receptors are necessary for influenza viruses when they will enter cells. To assess the susceptibility of pancreatic cells to influenza viruses, the surface location of SA receptors were observed in the mouse and human pancreatic cell lines PAN02 and PANC-1, respectively. Both α-2,3- and α-2,6-linked SA receptors were expressed on the surface of PAN02 and PANC-1 cells (Figures 1A,B). To quantitatively analyze the SA residues, pancreatic cells were stained with different concentrations of FITC-conjugated lectins and analyzed by flow cytometry. The results showed that expression of α-2,3-linked (FITC-MAA) and α-2,6-linked (FITC-SNA) SA receptors was detected in both PAN02 and PANC-1 cells at all lectin concentrations (Figure 1). The mean fluorescence intensity (MFI) increased with increasing concentrations of lectin. In the PAN02 cells, the MFI of SNA was dramatically higher (>4 fold) than that of MAA at each concentration tested (Figure 1C). However, PANC-1 cells exhibited higher MFI of MAA compared with SNA (Figure 1D). These results are consistent with the observations obtained by confocal microscopy and suggest that SA receptors are abundant on the surface of PAN02 and PANC-1 cells. Furthermore, *in vivo* results also showed the expression pattern of sialic acid receptors of mouse pancreatic cells and were consistent with above results of the of PAN02 and PANC-1 cell lines (Figure 1E). In summary, the results demonstrate that both α-2,3- and α-2,6-linked SA receptors are expressed on the surface of pancreatic cells.

### Pancreatic cells support H5N1 IAV replication

**Figure 2:**
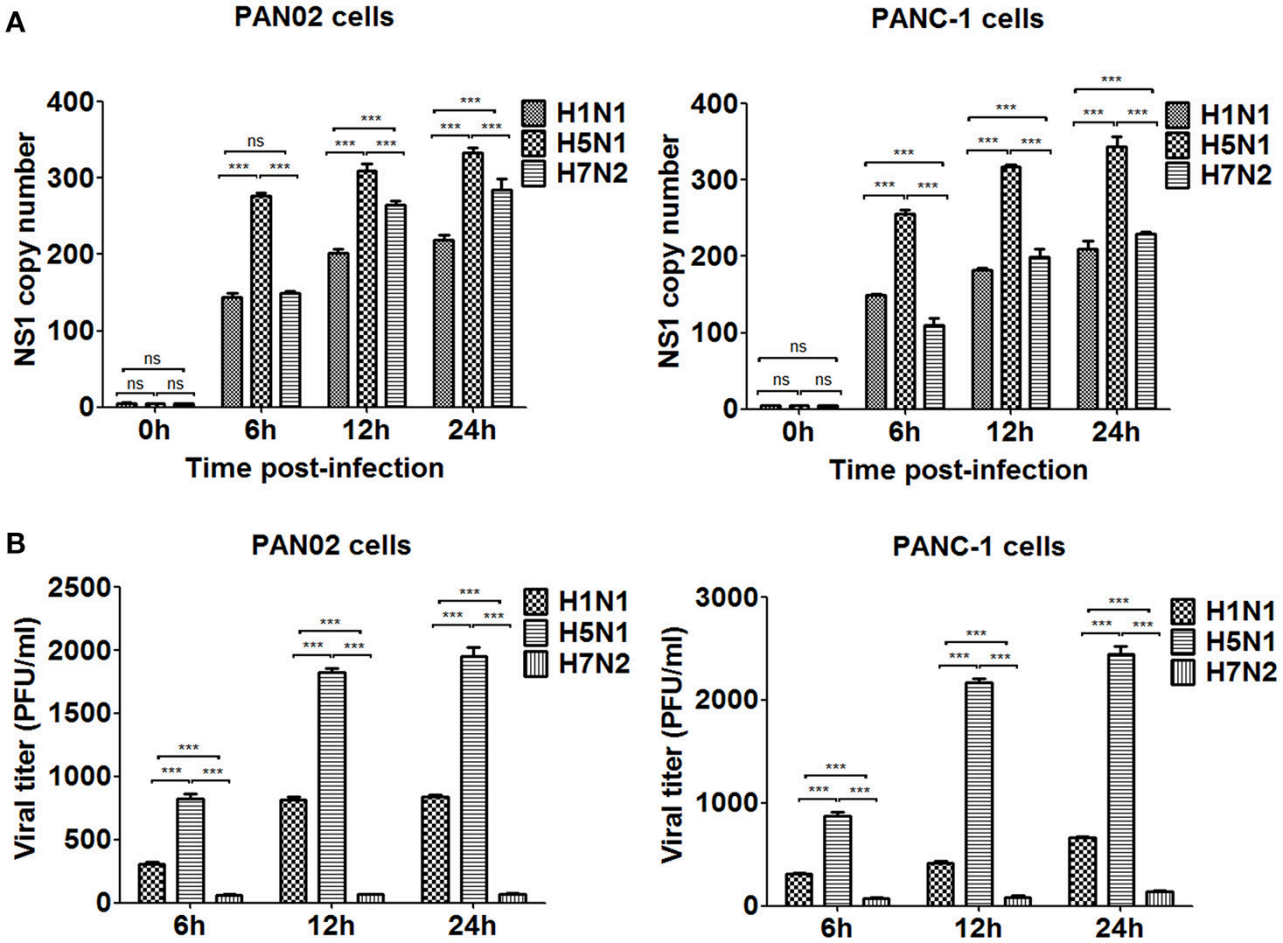
H5N1 IAV could productively infect pancreatic cells while H1N1 and H7N2 showed limited replication. PAN02 and PANC-1 cells were infected with the three IAV subtypes at an equal multiplicity of infection (MOI) of 1 for the periods specified. **(A)** Cells were homogenized in Trizol reagent and the viral non-structural protein 1 (NS1) gene was quantified using real time quantitative PCR (RT-qPCR). **(B)** The culture media supernatant was used to determine viral titers by plaque assay. The results shown here were pooled from three independent replicates (ns, not significantly and ^***^P < 0.001).

**Figure 3:**
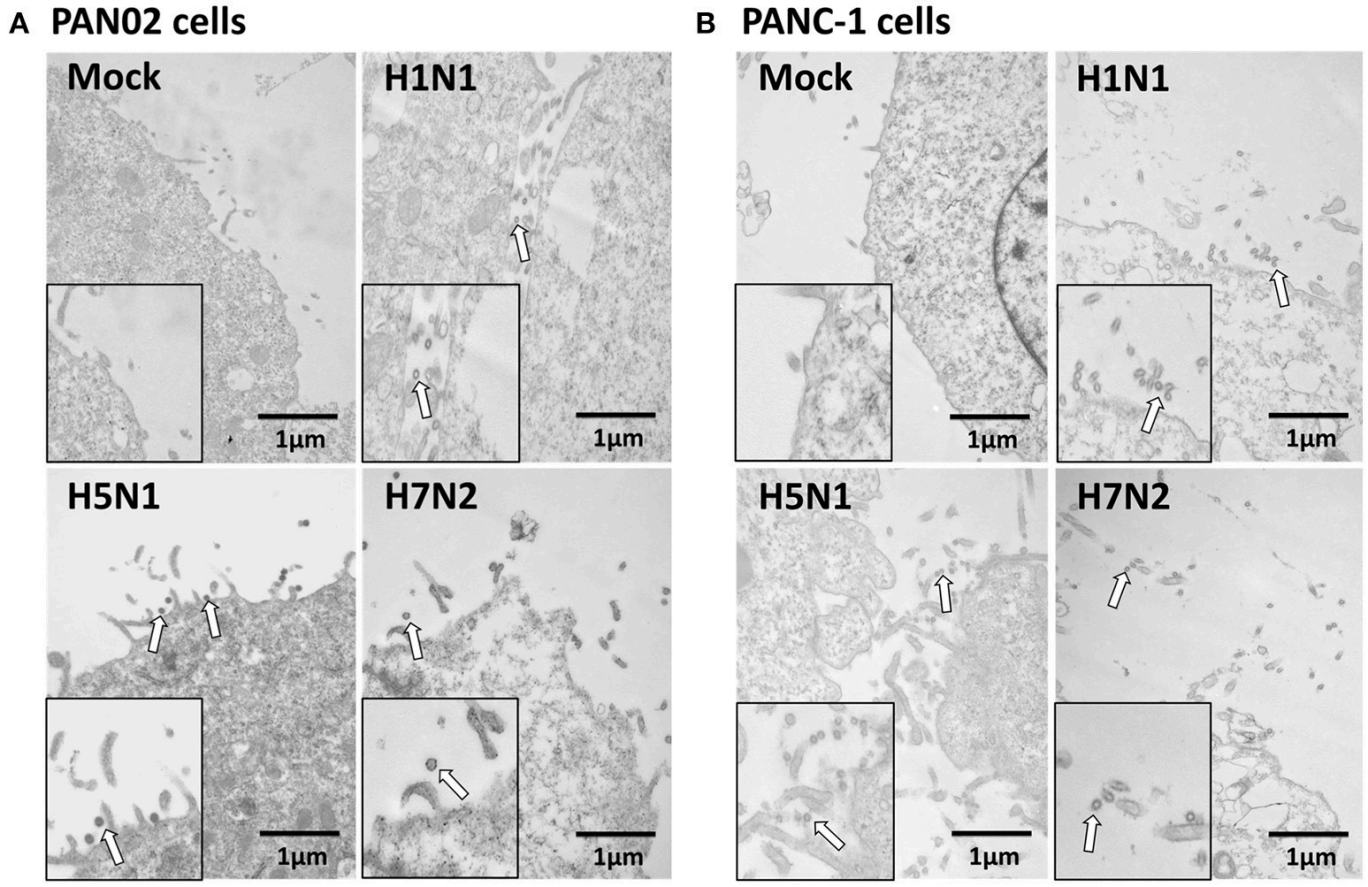
Pancreatic cells release infectious virus following H5N1 IAV infection. **(A)** PAN02 and **(B)** PANC-1 cells were mock-treated or infected with three IAV subtypes at an MOI of 1 and transmission electron microscopy was used to observe the viruses released from the cell surface. Arrows denote the virus particles.

**Figure 4:**
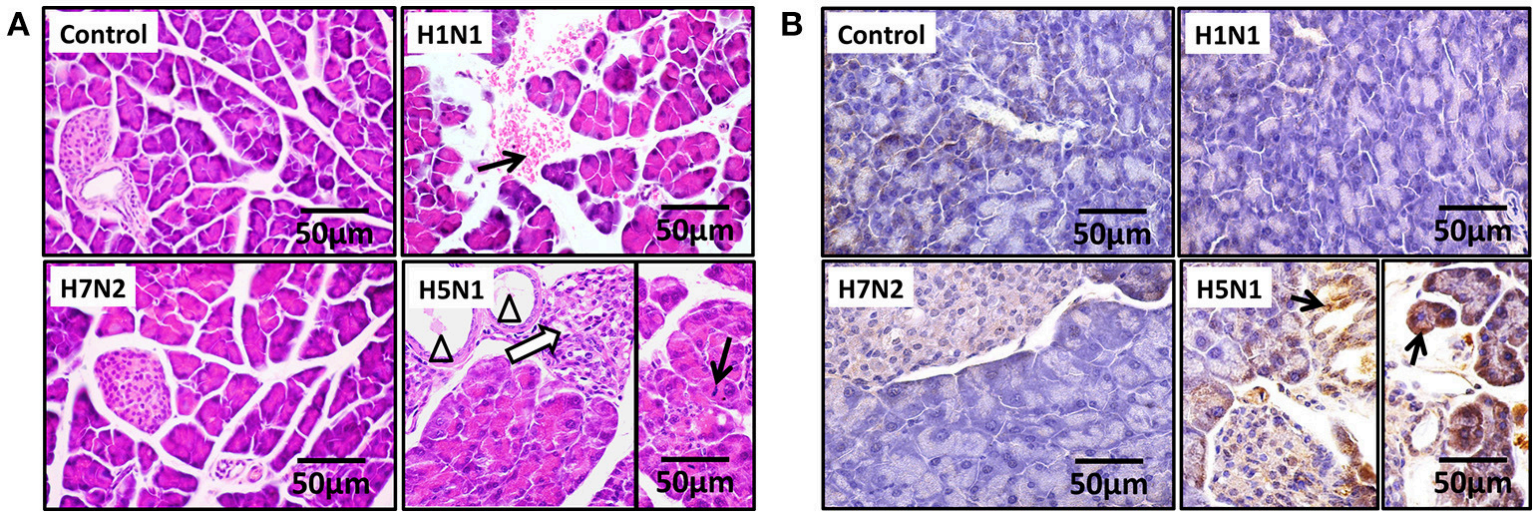
H5N1 IAV could infect pancreatic cells *in vivo*. Representative pancreas sections from control or IAV-infected mice. **(A)** Sections were analyzed by H & E staining. Black arrows indicate hemorrhage. Hollow arrows indicate necrosis of pancreatic cells. Triangles indicate dilatation and exudate in pancreatic duct. **(B)** Sections were analyzed by IHC staining. Black arrows indicate positive signals.

To assess if H5N1 IAV can replicate productively in pancreatic cells, we investigated the kinetics of IAV replication in PAN02 and PANC-1 cells by viral RNA expression and plaque formation. As shown in Figure 2A, the levels of the viral NS1 gene could be detected by RT-qPCR in both cell types following infection. Moreover, replication of the H5N1 virus was the most efficient among the three IAV subtypes. Besides, the viral infectivity titers in both PAN02 cells and PANC-1 cells treated with H5N1 were higher than those treated with H1N1 by the method of plaque assay. H7N2 seemed not to be replicated in both cells, which detected by plaque assay (Figure 2B). To further validate the permissiveness of PAN02 and PANC-1 cells for H5N1 IAV replication, we observed the cells using TEM. As shown in Figure 3, budding virions were found on the surface of both pancreatic cell types infected with IAV including H1N1, H5N1 and H7N2. Furthermore, much more viral particles were seen on the surface of H1N1 or H5N1 infected pancreatic cells than H7N2 infected cells. To further assess whether IAVs could infect pancreatic cells, *in vivo* experiments were performed. Mice were treated with H1N1, H5N1, H7N2, or PBS. The pancreas tissues were collected and analyzed by histopathological and immunohistochemical staining. As shown in Figure 4A, no pathological damage was found in the pancreas of PBS or H7N2 treated group. The mice in H1N1 group showed slight changes such as hemorrhage. In contrast, more serious injury was found in H5N1 group, including dilatation and exudate in pancreatic duct and necrosis of pancreatic cells. To detect the distribution of viral antigen in the pancreas, immunohistochemical staining was performed using a specific IAV nucleoprotein (NP) antibody (Figure 4B). Positive signals were widely found in both pancreatic duct epithelial cells and pancreatic cells in H5N1 group. These results indicate that H5N1 IAVs could productively infect pancreatic cells while H1N1 and H7N2 showed limited replication.

### H5N1 IAV infection-induced apoptosis of pancreatic cells

**Figure 5:**
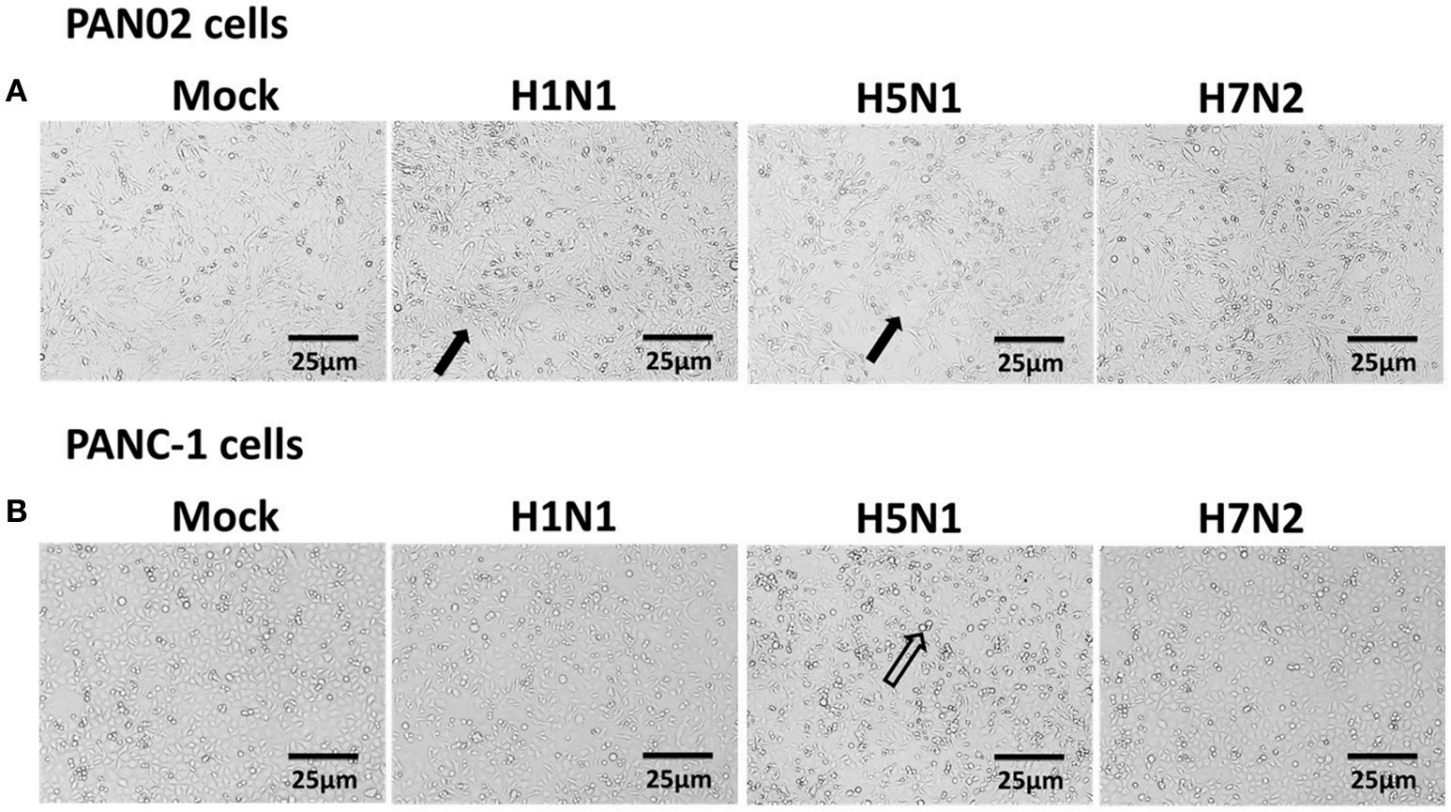
Cytopathic effects were induced in H5N1 IAV-infected pancreatic cells. **(A)** PAN02 and **(B)** PANC-1 cells were mock-treated or infected with H1N1, H5N1, or H7N2 at an MOI of 1 for 12 h. Morphologic analysis of the effect of IAV infection on cell death were taken. Cell morphology was visualized by light microscopy. Black arrows indicate numerous rounded cells. Hollow arrows indicate dead cells. Bar = 25 μm.

**Figure 6:**
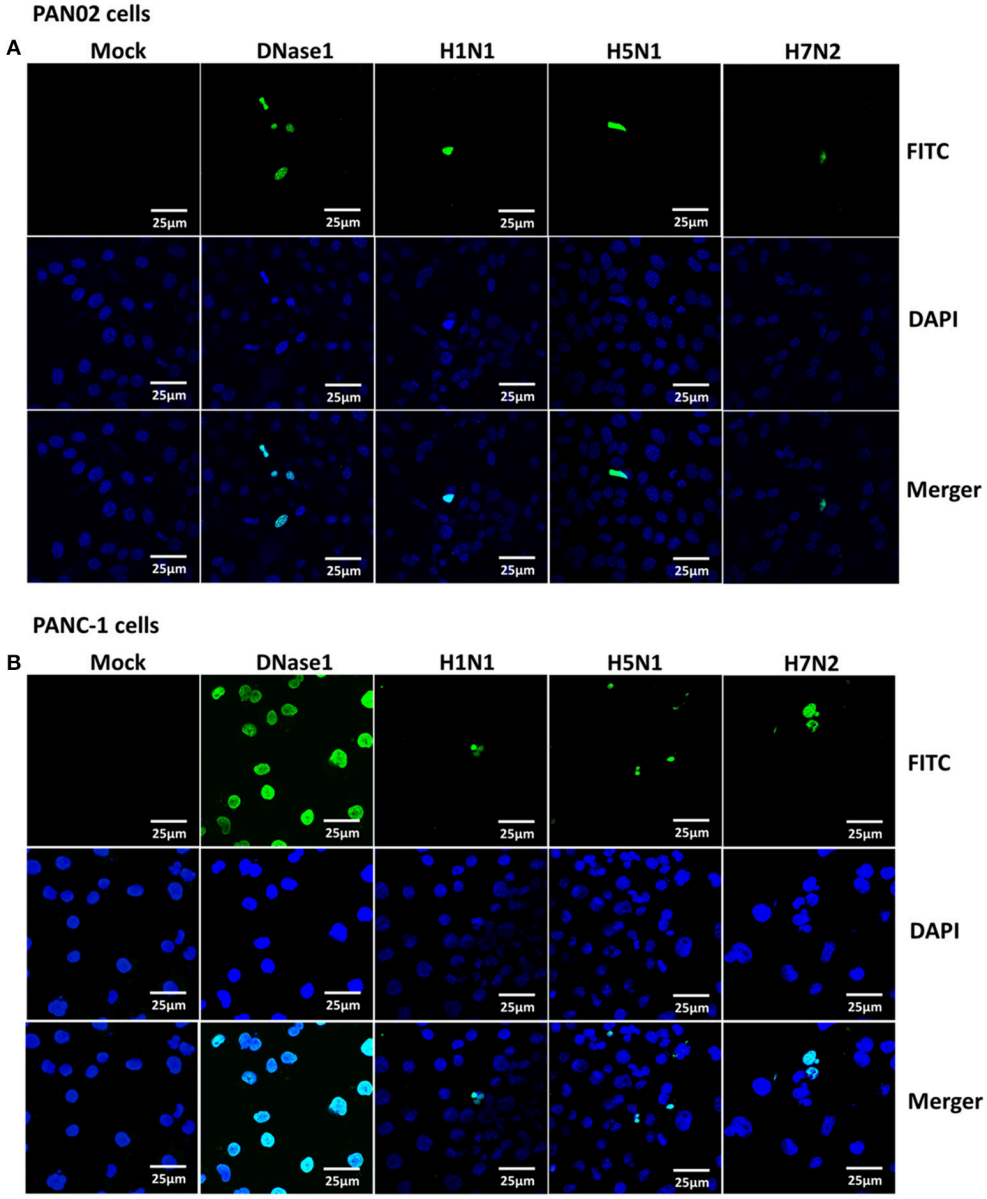
Apoptosis was induced in H5N1 IAV-infected pancreatic cells. **(A)** PAN02 and **(B)** PANC-1 cells were mock-treated or infected with H1N1, H5N1, or H7N2 at an MOI of 1 for 12 h. A terminal deoxynucleotidyl transferase-mediated dUTP-biotin nick end-labeling (TUNEL) assay was used to measure apoptosis in PAN02 and PANC-1 cells with DNase I as a positive control. Blue showed nucleus and green showed positive TUNEL signals.

Extensive cytopathic effects were observed in PAN02 and PANC-1 cells during H5N1 IAV replication. While subtle pathologies were detected in cells following H1N1 infection and no obvious pathologies were shown in H7N2 group (Figure 5). To assess whether apoptosis would occur following IAV infection in pancreatic cells, *in situ* TUNEL staining was employed, which detect DNA strand breakage. Apoptosis occurred in H5N1, H1N1, or H7N2 infected PAN02 and PANC-1 cells but not in mock-treated cells. DNase I was used as positive control (Figure 6).

**Figure 7:**
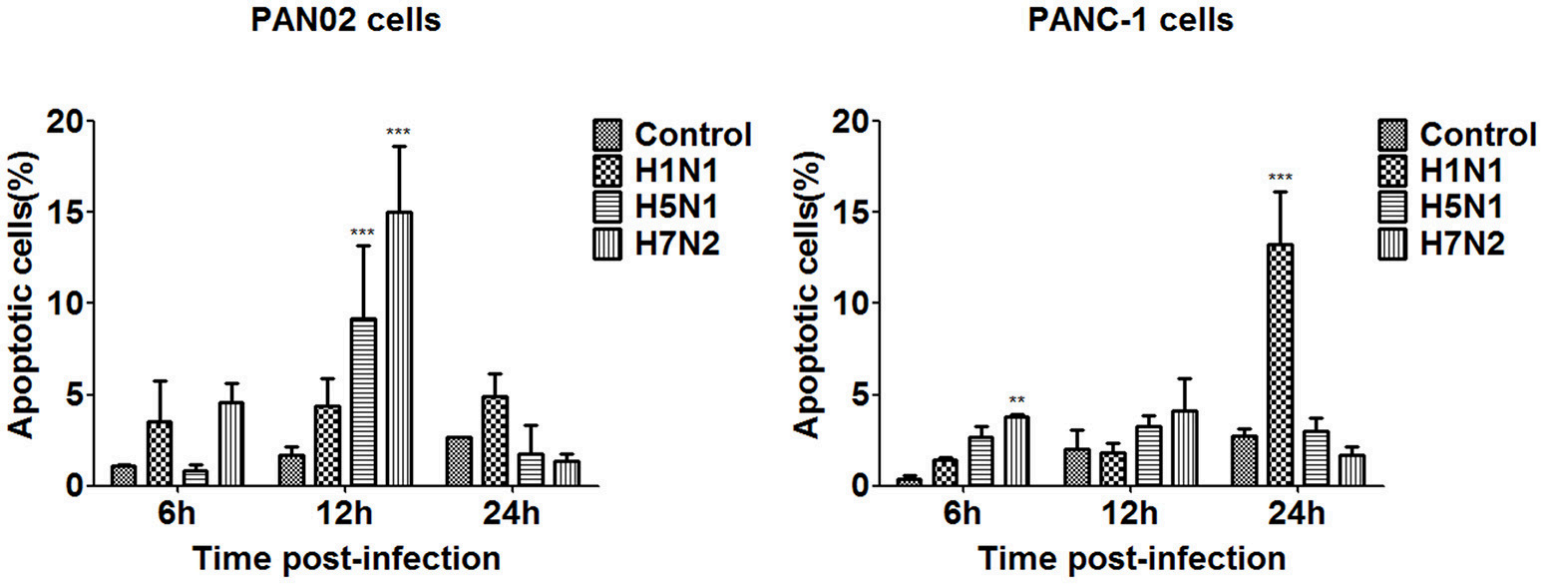
Pancreatic cell apoptosis was measured at specified times following IAV infection. PAN02 and PANC-1 cells were mock-treated or infected with IAV (H1N1, H5N1, or H7N2) at an MOI of 1, taxol served as a positive control. Then the cells were harvested at 6, 12, and 24 h post-infection. The total number of apoptotic cells in early stage was analyzed by flow cytometry. Asterisks indicate statistically significantly values compared with mock-treated cells (^**^P < 0.01, and ^***^P < 0.001).

The dynamic change of apoptosis was then measured in virus infected PAN02 and PANC-1 cells at designated times following infection. FITC-conjugated annexin V and propidium iodide double staining were processed and analyzed by flow cytometry. As shown in Figure 7, apoptosis occurred slightly 6 h following infection. Among the three IAV subtypes, the proportion of apoptosis in H5N1-infected and H7N2-infected cells was the highest 12 h following infection, but subsequently decreased to a level lower than mock-treated cells at 24 h. However, apoptosis in H1N1-infected cells occurred in a time-dependent manner, increasing over the course of the experiment. Together, these results suggest that the H5N1 can induce the apoptotic response in pancreatic cells.

### H5N1 IAV induced cytokine and chemokine production in pancreatic cells

**Figure 8:**
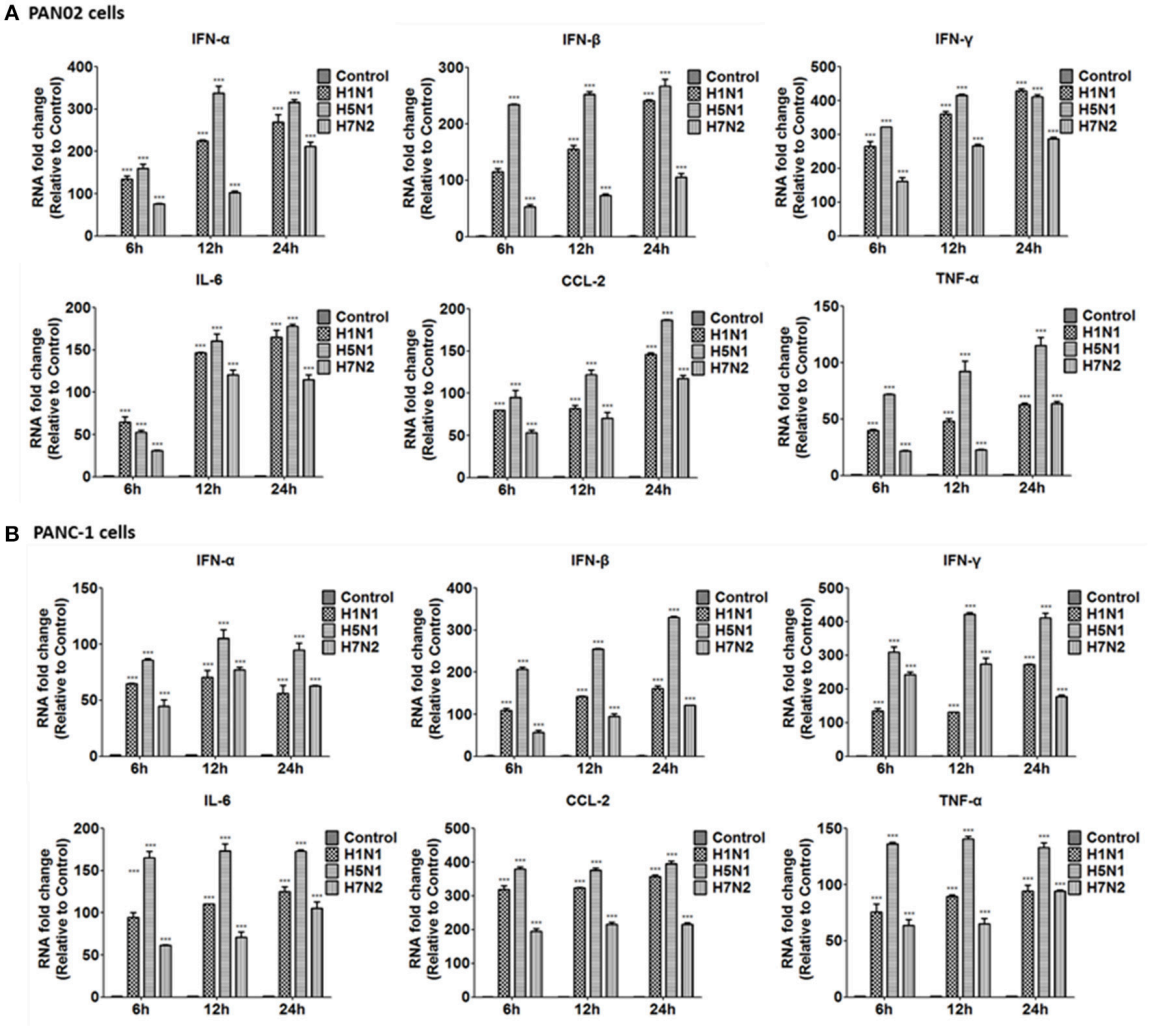
H5N1 IAV infection increased the release of pro-inflammatory cytokines and chemokines outside the cells to function. **(A)** PAN02 and **(B)** PANC-1 cells were treated or infected as described in Figure 5 and the culture media supernatant were harvested at 6, 12, and 24 h post-infection. The expression of interferon (IFN)-α, IFN-β, IFN-γ, chemokine (C-C motif) ligand 2 (CCL2), and tumor necrosis factor (TNF)-α was analyzed by RT-qPCR. Graphs shown are the mean ± SD of three independent replicates. Asterisks indicate statistically significant increases compared to mock-treated cells (ns, not significantly and ^***^P < 0.001).

**Figure 9:**
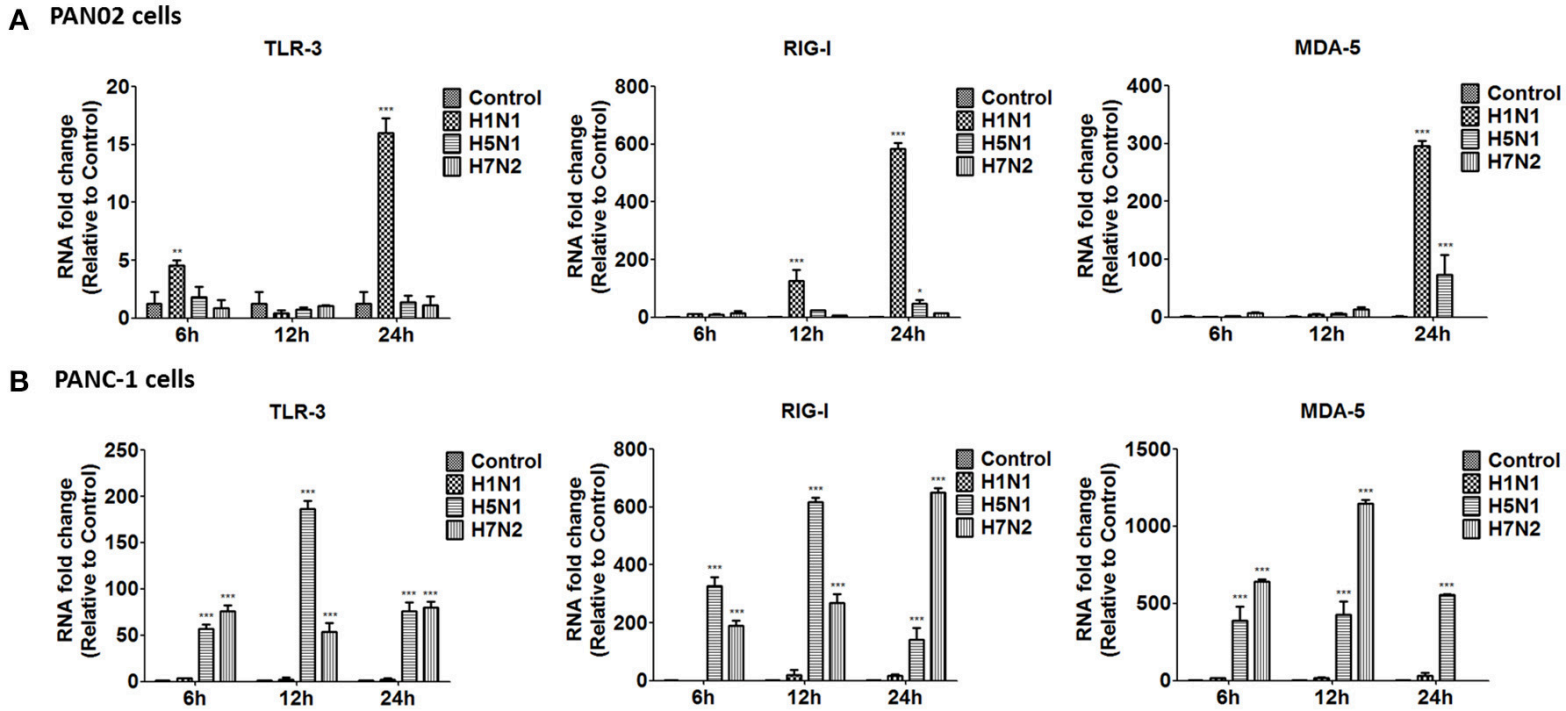
H5N1 IAV upregulates the mRNA expression of Toll-like receptor 3 (TLR3), cytolytic RNA helicases retinoic acid-inducible gene I (RIG-I), and melanoma differentiation-associated gene 5 (MDA-5). **(A)** PAN02 and **(B)** PANC-1 cells were treated or infected as described in Figure 5 and harvested at 6, 12, and 24 h post-infection. Total RNA was isolated at the designated times and examined by RT-qPCR. The mRNA expression of TLR3, RIG-I, and MDA-5 is shown. The data are presented as the relative fold change over mock treatment. Graphs shown are the mean ± standard deviation (SD) of three independent replicates. Asterisks indicate statistically significant increases compared with mock-treated cells (^*^P < 0.05, ^**^P < 0.01, and ^***^P < 0.001).

To further investigate the inflammatory response induced by H5N1 influenza infection in PAN02 and PANC-1 cells, the mRNA expression profiles of IFN-α, IFN-β, IFN-γ, IL-6, CCL-2, and TNF-α were measured by RT-qPCR. The production of a great amount of pro-inflammatory cytokines and chemokines in pancreatic cells was upregulated during viral infection (Figure 8). Among three subtypes of IAVs, H5N1 could significantly cause two pancreatic cells to produce a robust inflammatory response and release much more cytokines and chemokines. In short, these results suggest that pancreatic cells participate in the inflammatory response by producing various pro-inflammatory cytokines and chemokines during IAV infection. Since the PRRs serve as a crucial actor in the regulation of the body's immune response during IAV infection, we examined the expression of several viral RNA sensors TLR3, RIG-I, and MDA-5, which are involved in the transduction of inflammatory signals (Figure 9). The mRNA expression levels of these sensors in PAN02 cells infected with H1N1 was the highest among the three IAV subtypes, peaking at 24 h following infection (Figure 9A). However, H5N1 and H7N2 could also activate signaling pathways in PANC-1 cells (Figure 9B). These data indicate that the TLR3, RIG-I, and MDA-5 signaling pathways are involved in pancreatic cells following H5N1 IAV infection.

## Discussion

The aim of this study was to evaluate H5N1 IAV replication in pancreatic cells from two sources and to evaluate the consequences of infection at the molecular and cellular level *in vitro*. We were able to demonstrate that H5N1 IAV can replicate, induce apoptosis, and stimulate the release of inflammatory cytokines in two different pancreatic cancer cell lines. Despite previous studies that reported pancreatic injury induced by IAV infenction in both animals and humans as well as the research performed by Capua et al., which has attempted to establish whether IAV can grow in pancreatic cells, to date the molecular mechanism of H5N1 IAV pathogenesis in the pancreas was poorly reported. Our study has generated novel *in vitro* and *in vivo* data which suggested that H5N1 IAV could grow in both mouse and human pancreatic cell lines and induce apoptosis as well as the pro-inflammatory cytokine response.

Attachment to SA receptors, which locate on the surface of cells, is necessary for IAV infection. Here we have showed that both α-2,3- and α-2,6-linked SA receptors were located on the surface of PAN02 and PANC-1 cells. Although Kasloff et al. (Kasloff et al., 2014)described the expression of receptors on some pancreatic cancer cell lines including PANC-1 cells and demonstrated that these cells contain certain levels of α-2,3- and α-2,6-linked SA receptors, we have provided the first description of differences in the expression of SA receptors between mouse PAN02 and human PANC-1 cells. To the best of our knowledge, we firstly showed that SA receptors were expressed on the surface of cells originating from the mouse pancreas. In particular, PAN02 cells usually contained higher levels of α-2,6- than α-2,3-linked SA receptors, rendering the cells easy to attachment by human influenza viruses, while PANC-1 cells were more easily infected by avian influenza viruses as a result of relatively higher levels of α-2,3-linked SA receptors.

We have also revealed that the H5N1 IAV can infect and replicate in PAN02 and PANC-1 cells productively. This was evident with the replication kinetics experiments, although we believe the TEM images were the most compelling. In contrast with the research of Capua et al. (2013) and Kasloff et al. (2014) in which only human pancreatic cells were established to suggest the IAV replication, the infection of both PAN02 and PANC-1 cells with IAV in this study provided further evidence of the replication ability of H5N1 IAV in pancreatic cells of mouse and human origin. Notably, our studies suggest that pancreatic cells can support H1N1 and H7N2 IAV replication, but that the life cycle of the virus is impeded in cells. *In vivo* experiments further demonstrated that only H5N1 IAV had the positive pathogenicity and infectivity in the pancreas, rather than H7N2 and H1N1. Previous studies have suggested that a deficiency in essential factors supporting productive infections, intrinsic antiviral immunity that dramatically restrict the viruses replicating productively could explain the limited release of the virus (Yan and Chen, 2012; Marcet et al., 2013). These will be avenues for us to explore the mechanism involved in H5N1 IAV-infected pancreatic cells.

Multiple factors can induce the apoptosis. Different IAV strains can induce various apoptosis, which is also related to the cell type. In the present study, three IAV subtypes, H1N1, H5N1, and H7N2, were used to confirm induction of apoptosis in PAN02 and PANC-1 cells with several assays, including the observation of typical morphological features using positive TUNEL staining and flow cytometric quantitative analysis. In contrast with the results of Capua et al. (2013), which only showed that human PANC-1 cells could undergo apoptosis following influenza viral infection, we firstly showed that induction of apoptosis by IAV challenge in pancreatic cells lines derived from both mouse and human. Several apoptotic signaling pathways involved in influenza virus infection have been well reported (Takizawa et al., 1995; Balachandran et al., 2000; Elmore, 2007; Brincks et al., 2008; Xing et al., 2011), which associated with cell type and viral components. The molecular mechanisms underlying apoptosis in H5N1 IAV-infected pancreatic cells are not well understood and thus will require further exploration in future studies.

In IAV infection, the role of PRRs, including TLRs and RLRs, is indispensable and activated intracellular pathways contribute to the releasing of crucial pro-inflammatory cytokines and chemokines in several types of IAV-infected cells, including alveolar epithelial cells. Here, we have provided evidence that the TLR3, RIG-I, and MDA5 signaling pathways are activated in pancreatic cells following H5N1 infection. In addition, a previous report detailed that IAV infection could trigger a robust pro-inflammatory response in human pancreatic islet cells, which caused severe damage of the islets (Capua et al., 2013). Here, we have demonstrated that H5N1 IAV can infect PAN02 and PANC-1 cells located in the exocrine pancreas, which resulted in the expression of several cytokines and chemokines, including a remarkable upregulation in the levels of IFN-α, IFN-β, IFN-γ, CCL-2, IL-6, and TNF-α, which deeply involved in host inflammatory response and pancreatic injury(Sarraf, 1994; Hierholzer et al., 1998; Lampasona et al., 2003; Perrone et al., 2010; Mehmeti et al., 2011; Tono et al., 2015). Additionally, our results here show that H5N1, H1N1 and H7N2 could induce different cytokine and chemokine kinetics in pancreatic cells. The magnitude of inflammatory responses caused by H5N1 was greater than H1N1 and H7N2 in both PAN02 cells and PANC-1 cells. Notably, it seems to have a positive correlation between NS1 copy number and cytokine and chemokine expressions, which suggests that viral replication appears to accelerate the secreting of pro-inflammatory cytokines and chemokines in H5N1-infected pancreatic cells. Thus, we have provided novel evidence that pancreatic cells may be involved in the inflammatory and antiviral responses to H5N1 IAV infection. Previous studies have shown that inflammatory cytokine storm during IAV infection and apoptosis have an interactive relation. Hyper-production of cytokines may promote apoptosis of pulmonary epithelial cells and certain immunocytes (Li et al., 2007; Hu et al., 2012). Moreover, virus-induced apoptosis could also promote the secretions of pro-inflammatory cytokines and chemokines (Liu et al., 2014). In our present study, the results also showed the promoting relationship between apoptosis and cytokine and chemokine expressions in PAN02 and PANC-1 cells during H5N1 IAV infection. Herein, our results further demonstrate that over expression of inflammatory cytokines caused by virus infection and apoptosis have an important role in the pathogenesis of IAV and are intrinsically linked.

In summary, our present data suggest that pancreatic cells are susceptible to H5N1 IAV infection and that virus-induced apoptosis and over expression of pro-inflammatory cytokines and chemokines occur in pancreatic cells during H5N1 IAV infection. Considering the potential role of IAV infection in the etiopathogenesis of pancreatic injury in mammals, this study provides novel ideas and may benefit the development of new therapeutic strategy against H5N1 IAV infection.

## Author contributions

CH, SZ, KX, YH, MW, and HT: conceived and designed the experiments. CH, KX, YT, YH, MW, PQ, and JX: performed the experiments. CH, KX, and YH: analyzed the data. CH, KX, and YH: contributed reagents, materials, analysis tools. CH and YH: wrote the paper. All authors reviewed the manuscript.

### Conflict of interest statement

The authors declare that the research was conducted in the absence of any commercial or financial relationships that could be construed as a potential conflict of interest. The reviewer BB and handling editor declared their shared affiliation at time of review.

## References

[B1] Balachandran S. Roberts P. C. Kipperman T. Bhalla K. N. Compans R. W. Archer D. R.. (2000). Alpha/beta interferons potentiate virus-induced apoptosis through activation of the FADD/Caspase-8 death signaling pathway. J. Virol. 74, 1513–1523. 10.1128/JVI.74.3.1513-1523.2000 10627563 PMC111487

[B2] Brincks E. L. Kucaba T. A. Legge K. L. Griffith T. S. (2008). Influenza-induced expression of functional tumor necrosis factor-related apoptosis-inducing ligand on human peripheral blood mononuclear cells. Hum. Immunol. 69, 634–646. 10.1016/j.humimm.2008.07.012 18723061 PMC2597454

[B3] Brydon E. W. Morris S. J. Sweet C. (2005). Role of apoptosis and cytokines in influenza virus morbidity. FEMS Microbiol. Rev. 29, 837–850. 10.1016/j.femsre.2004.12.003 16102605

[B4] Calore E. E. Uip D. E. Perez N. M. (2011). Pathology of the swine-origin influenza A (H1N1) flu. Pathol. Res. Pract. 207, 86–90. 10.1016/j.prp.2010.11.003 21176866

[B5] Capua I. Mercalli A. Pizzuto M. S. Romero-Tejeda A. Kasloff S. De Battisti C.. (2013). Influenza A viruses grow in human pancreatic cells and cause pancreatitis and diabetes in an animal model. J. Virol. 87, 597–610. 10.1128/JVI.00714-12 23097451 PMC3536404

[B6] Chaves A. J. Vergara-Alert J. Busquets N. Valle R. Rivas R. Ramis A.. (2014). Neuroinvasion of the highly pathogenic influenza virus H7N1 is caused by disruption of the blood brain barrier in an avian model. PLoS ONE 9:e115138. 10.1371/journal.pone.0115138 25506836 PMC4266681

[B7] Cheng X. W. Lu J. Wu C. L. Yi L. N. Xie X. Shi X. D.. (2011). Three fatal cases of pandemic 2009 influenza A virus infection in Shenzhen are associated with cytokine storm. Respir. Physiol. Neurobiol. 175, 185–187. 10.1016/j.resp.2010.11.004 21075220

[B8] Cheung C. Y. Poon L. L. Lau A. S. Luk W. Lau Y. L. Shortridge K. F.. (2002). Induction of proinflammatory cytokines in human macrophages by influenza A (H5N1) viruses: a mechanism for the unusual severity of human disease? Lancet 360, 1831–1837. 10.1016/S0140-6736(02)11772-7 12480361

[B9] Cillóniz C. Shinya K. Peng X. Korth M. J. Proll S. C. Aicher L. D.. (2009). Lethal influenza virus infection in macaques is associated with early dysregulation of inflammatory related genes. PLoS Pathog. 5:e1000604. 10.1371/journal.ppat.1000604 19798428 PMC2745659

[B10] de Jong M. D. Simmons C. P. Thanh T. T. Hien V. M. Smith G. J. Chau T. N.. (2006). Fatal outcome of human influenza A (H5N1) is associated with high viral load and hypercytokinemia. Nat. Med. 12, 1203–1207. 10.1038/nm1477 16964257 PMC4333202

[B11] Elmore S. (2007). Apoptosis: a review of programmed cell death. Toxicol. Pathol. 35, 495–516. 10.1080/01926230701320337 17562483 PMC2117903

[B12] Haider N. Sturm-Ramirez K. Khan S. U. Rahman M. Z. Sarkar S. Poh M. K.. (2017). Unusually high mortality in waterfowl caused by highly pathogenic avian influenza A(H5N1) in Bangladesh. Transbound. Emerg. Dis. 64, 144–156. 10.1111/tbed.12354 25892457 PMC4635058

[B13] Herold S. Ludwig S. Pleschka S. Wolff T. (2012). Apoptosis signaling in influenza virus propagation, innate host defense, and lung injury. J. Leukoc. Biol. 92, 75–82. 10.1189/jlb.1011530 22345705

[B14] Hierholzer C. Kalff J. C. Omert L. Tsukada K. Loeffert J. E. Watkins S. C.. (1998). Interleukin-6 production in hemorrhagic shock is accompanied by neutrophil recruitment and lung injury. Am. J. Physiol. 275(3 Pt 1), L611–L621. 9728057 10.1152/ajplung.1998.275.3.L611

[B15] Hu Y. Jin Y. Han D. Zhang G. Cao S. Xie J.. (2012). Mast cell-induced lung injury in mice infected with H5N1 influenza virus. J. Virol. 86, 3347–3356. 10.1128/JVI.06053-11 22238293 PMC3302317

[B16] Huo C. Zhang S. Zhang S. Wang M. Qi P. Xiao J.. (2017). Mice with type 1 diabetes exhibit increased susceptibility to influenza A virus. Microb. Pathog.. 10.1016/j.micpath.2017.10.026 29066377

[B17] Ito T. Kobayashi Y. Morita T. Horimoto T. Kawaoka Y. (2002). Virulent influenza A viruses induce apoptosis in chickens. Virus Res. 84, 27–35. 10.1016/S0168-1702(01)00414-2 11900836

[B18] Kasloff S. B. Pizzuto M. S. Silic-Benussi M. Pavone S. Ciminale V. Capua I. (2014). Oncolytic activity of avian influenza virus in human pancreatic ductal adenocarcinoma cell lines. J. Virol. 88, 9321–9334. 10.1128/JVI.00929-14 24899201 PMC4136238

[B19] Lampasona V. Rio J. Franciotta D. Furlan R. Avolio C. Fazio R.. (2003). Serial immunoprecipitation assays for interferon–(IFN)-beta antibodies in multiple sclerosis patients. Eur. Cytokine Netw. 14, 154–157. 14656689

[B20] Leon'teva G. F. Varechkova E. Dubrovina T. Ia. Ivanova I. A. Shidlovskaia N. K. (1989). [Characteristic features of experimental influenza in mice–long-term presence of viral antigens in the spleen]. *Vestn Akad Med Nauk SSSR* (11), 87–92. 2623939

[B21] Li X. McKinstry K. K. Swain S. L. Dalton D. K. (2007). IFN-gamma acts directly on activated CD4+ T cells during mycobacterial infection to promote apoptosis by inducing components of the intracellular apoptosis machinery and by inducing extracellular proapoptotic signals. J. Immunol. 179, 939–949. 10.4049/jimmunol.179.2.939 17617585 PMC2532516

[B22] Liu B. Meng D. Wei T. Zhang S. Hu Y. Wang M. (2014). Apoptosis and pro-inflammatory cytokine response of mast cells induced by influenza A viruses. PLoS ONE 9:e100109. 10.1371/journal.pone.0100109 24923273 PMC4055757

[B23] López Cde H. Roca R. F. Daunis J. V. (2009). [Pneumonia and the acute respiratory distress syndrome due to influenza A (H1N1) virus]. Med. Intensiva 33, 455–458. 10.1016/j.medin.2009.09.004 19854543

[B24] Marcet C. W. St Laurent C. D. Moon T. C. Singh N. Befus A. D. (2013). Limited replication of influenza A virus in human mast cells. Immunol. Res. 56, 32–43. 10.1007/s12026-012-8377-4 23055084

[B25] Matrosovich M. N. Matrosovich T. Y. Gray T. Roberts N. A. Klenk H. D. (2004). Human and avian influenza viruses target different cell types in cultures of human airway epithelium. Proc. Natl. Acad. Sci. U.S.A. 101, 4620–4624. 10.1073/pnas.0308001101 15070767 PMC384796

[B26] Mehmeti I. Lenzen S. Lortz S. (2011). Modulation of Bcl-2-related protein expression in pancreatic beta cells by pro-inflammatory cytokines and its dependence on the antioxidative defense status. Mol. Cell. Endocrinol. 332, 88–96. 10.1016/j.mce.2010.09.017 20933054

[B27] Meng D. Huo C. Wang M. Xiao J. Liu B. Wei T.. (2016). Influenza A viruses replicate productively in mouse mastocytoma cells (P815) and trigger pro-inflammatory cytokine and chemokine production through TLR3 signaling pathway. Front. Microbiol. 7:2130. 10.3389/fmicb.2016.02130 28127293 PMC5226950

[B28] Ouyang L. Shi Z. Zhao S. Wang F. T. Zhou T. T. Liu B.. (2012). Programmed cell death pathways in cancer: a review of apoptosis, autophagy and programmed necrosis. Cell Prolif. 45, 487–498. 10.1111/j.1365-2184.2012.00845.x 23030059 PMC6496669

[B29] Pantin-Jackwood M. J. Swayne D. E. (2007). Pathobiology of Asian highly pathogenic avian influenza H5N1 virus infections in ducks. Avian Dis. 51, 250–259. 10.1637/7710-090606R.1 17494561

[B30] Perrone L. A. Szretter K. J. Katz J. M. Mizgerd J. P. Tumpey T. M. (2010). Mice lacking both TNF and IL-1 receptors exhibit reduced lung inflammation and delay in onset of death following infection with a highly virulent H5N1 virus. J. Infect. Dis. 202, 1161–1170. 10.1086/656365 20815704 PMC2941567

[B31] Raman R. Tharakaraman K. Shriver Z. Jayaraman A. Sasisekharan V. Sasisekharan R. (2014). Glycan receptor specificity as a useful tool for characterization and surveillance of influenza A virus. Trends Microbiol. 22, 632–641. 10.1016/j.tim.2014.07.002 25108746 PMC4252848

[B32] Reperant L. A. van de Bildt M. W. van Amerongen G. Leijten L. M. Watson S. Palser A.. (2012). Marked endotheliotropism of highly pathogenic avian influenza virus H5N1 following intestinal inoculation in cats. J. Virol. 86, 1158–1165. 10.1128/JVI.06375-11 22090101 PMC3255817

[B33] Sarraf C. (1994). Tumor-necrosis-factor and cell-death in tumors (review). Int. J. Oncol. 5, 1333–1339. 21559718 10.3892/ijo.5.6.1333

[B34] Sharkova T. V. Potapova O. V. Shkurupy V. A. Shestopalov A. M. Drozdov I. G. Shestopalova L. V. (2008). Structural changes in the liver of mice infected with avian influenza virus subtype H5N1. Bull. Exp. Biol. Med. 146, 243–245. 10.1007/s10517-008-0262-8 19145328

[B35] Song F. Yu X. Zhong T. Wang Z. Meng X. Li Z.. (2018). Caspase-3 inhibition attenuates the cytopathic effects of EV71 infection. Front. Microbiol. 9:817. 10.3389/fmicb.2018.00817 29755438 PMC5932146

[B36] Suzuki Y. Ito T. Suzuki T. Holland R. E. Jr. Chambers T. M. Kiso M.. (2000). Sialic acid species as a determinant of the host range of influenza A viruses. J. Virol. 74, 11825–11831. 10.1128/jvi.74.24.11825-11831.2000 11090182 PMC112465

[B37] Takeuchi O. Akira S. (2009). Innate immunity to virus infection. Immunol. Rev. 227, 75–86. 10.1111/j.1600-065X.2008.00737.x 19120477 PMC5489343

[B38] Takizawa T. Fukuda R. Miyawaki T. Ohashi K. Nakanishi Y. (1995). Activation of the apoptotic Fas antigen-encoding gene upon influenza virus infection involving spontaneously produced beta-interferon. Virology 209, 288–296. 10.1006/viro.1995.1260 7539967

[B39] Tang Y. Lu J. Wu P. Liu Z. Tian Z. Zha G.. (2014). Inactivated vaccine with adjuvants consisting of pattern recognition receptor agonists confers protection against avian influenza viruses in chickens. Vet. Microbiol. 172, 120–128. 10.1016/j.vetmic.2014.05.007 24894132 PMC4208718

[B40] Tang Y. Wang Z. Huo C. Guo X. Yang G. Wang M.. (2018). Antiviral effects of Shuanghuanglian injection powder against influenza A virus H5N1 *in vitro* and *in vivo*. Microb. Pathog. 121, 318–324. 10.1016/j.micpath.2018.06.004 29864534

[B41] Tisoncik J. R. Korth M. J. Simmons C. P. Farrar J. Martin T. R. Katze M. G. (2012). Into the eye of the cytokine storm. Microbiol. Mol. Biol. Rev. 76, 16–32. 10.1128/MMBR.05015-11 22390970 PMC3294426

[B42] Tono T. Aihara S. Hoshiyama T. Arinuma Y. Nagai T. Hirohata S. (2015). Effects of anti-IL-6 receptor antibody on human monocytes. Mod. Rheumatol. 25, 79–84. 10.3109/14397595.2014.914016 24842475

[B43] Us D. (2008). [Cytokine storm in avian influenza]. Mikrobiyol. Bul. 42, 365–380. 18697437

[B44] Van Poucke S. G. Nicholls J. M. Nauwynck H. J. Van Reeth K. (2010). Replication of avian, human and swine influenza viruses in porcine respiratory explants and association with sialic acid distribution. Virol. J. 7, 38. 10.1186/1743-422X-7-38 20158900 PMC2829537

[B45] Xing Z. Harper R. Anunciacion J. Yang Z. Gao W. Qu B.. (2011). Host immune and apoptotic responses to avian influenza virus H9N2 in human tracheobronchial epithelial cells. Am. J. Respir. Cell Mol. Biol. 44, 24–33. 10.1165/rcmb.2009-0120OC 20118223 PMC3159084

[B46] Yan N. Chen Z. J. (2012). Intrinsic antiviral immunity. Nat. Immunol. 13, 214–222. 10.1038/ni.2229 22344284 PMC3549670

[B47] Yingst S. L. Saad M. D. Felt S. A. (2006). Qinghai-like H5N1 from domestic cats, northern Iraq. Emerging Infect. Dis. 12, 1295–1297. 10.3201/eid1208.060264 16972356 PMC3291230

[B48] Yu M. Levine S. J. (2011). Toll-like receptor, RIG-I-like receptors and the NLRP3 inflammasome: key modulators of innate immune responses to double-stranded RNA viruses. Cytokine Growth Factor Rev. 22, 63–72. 10.1016/j.cytogfr.2011.02.001 21466970 PMC3109132

[B49] Zhang S. Wei T. Tianv H. Cheng J. Xiao J. Wang M.. (2015). Small intestinal injury in mice infected with respiratory influenza A virus: evidence for virus induced gastroenteritis. Biotechnol. Lett. 37, 1585–1592. 10.1007/s10529-015-1847-8 25967033

