# Supplementary material for: H5N1 Influenza a Virus Replicates Productively in Pancreatic Cells and Induces Apoptosis and Pro-Inflammatory Cytokine Response
Source: Front Cell Infect Microbiol. 2018 Nov 6;8:386. doi: 10.3389/fcimb.2018.00386 (PMC6232254; doi:10.3389/fcimb.2018.00386)
Supplement: Supplementary file 1 [file Data_Sheet_1.PDF]

H5N1 influenza A virus replicate productively in pancreatic cells and induces apoptosis and pro-inflammatory cytokine response

Caiyun Huo, Kai Xiao, Shouping Zhang, Yuling Tang, Ming Wang, Peng Qi, Jin Xiao, Haiyan Tian, Yanxin Hu*

***Correspondence:** Yanxin Hu：huyx@cau.edu.cn

Table S1.

	Target name	Primers
Influenza A virus	NS1	5’- GCA ATT GGA ATC CTC ATC GG -3’
		5’- CAA CTC GTT TCG CCA TGT AGC -3’
Mouse	TLR3	5’- CCC TTC ACC TTT CCG -3’
		5’- TCA TCT AAG CCG TTG G -3’
	RIG-I	5’- TTC TAA AGC CTG GCA TAC TGA -3’
		5’- GCT ATC TCG TGC TCT TCC TC -3’
	MDA5	5’- CGA TCC GAA TGA TTG ATG CA -3’
		5’- AGT TGG TCA TTG CAA CTG CT -3’
	IFN-α	5’- AGC CTT GAC ACT CCT GGT ACA -3’
		5’- TGA GCC TTC TTG ATC TGC TG -3’
	IFN-β	5’- AGA TGT CCT CAA CTG CTC TC -3’
		5’- AGA TTC ACT ACC AGT CCC AG -3’
	IFN-γ	5’- GAC TGT GAT TGC GGG GTT GT -3’
		5’- GGC CCG GAG TGT AGA CAT CT -3’
	IL-6	5’- ACA GAA GGA GTG GCT AAG GA -3’
		5’- CGC ACT AGG TTT GCC GAG TA -3’
	CCL-2	5’- GCT TCT GGG CCT GCT GTT CA -3’
		5’- AGC TCT CCA GCC TAC TCA TT -3’
	TNF-α	5’- TCA CTG GAG CCT CGA ATG TC -3’
		5’- GTG AGG AAG GCT GTG CAT TG -3’
	β-actin	5’- GAG ACC TTC AAC ACC CCA GC -3’
		5’- ATG TCA CGC ACG ATT TCC C -3’
Human	TLR3	5’- AAA TTG GGC AAG AAC TCA CAG G -3’
		5’- GTG TTT CCA GAG CCG TGC TAA -3’
	RIG-I	5’- GAG CAG CAG GAT TCG AAG AGA -3’
		5’- TTG CTC TTC CTC TGC CTC TG -3’
	MDA5	5’- GAG GAA TCA GCA CGA GGA ATA A -3’
		5’- TCA GAT GGT GGG CTT TGA C -3’
	IFN-α	5’- ATG GCC TTG ACC TTT GCT TT -3’
		5’- TGG AAG ATT TCC TCA TAG C -3’
	IFN-β	5’- ATG ACC AAC AAG TGT CTC CTC CAA A -3’
		5’- TTC TTC CAG GAC TGT CTT CA -3’
	IFN-γ	5’- GGC ATT TTG AAG AAT TGG AAA G -3’
		5’- TTT GGA TGC TCT GGT CAT CTT -3’
	IL-6	5’- GCC CAG CTA TGA ACT CCT TCT -3’
		5’- GCG GCT ACA TCT TTG GAA TCT -3’
	CCL-2	5’- CTG CTC ATA GCA GCC ACC TT -3’
		5’- GCA CTG AGA TCT TCC TAT TGG TG -3’
	TNF-α	5’- CCA ACT GTC ACT CAT TGC TGA -3’
		5’- TTC CAA GAA GGA GAC CAT GTT T -3’
	GAPDH	5’- GCA AAT TCC ATG GCA CCG T -3’
		5’- TCG CCC CAC TTG ATT TTG G -3’
